# Notch Signaling Change in Pulmonary Vascular Remodeling in Rats with Pulmonary Hypertension and Its Implication for Therapeutic Intervention

**DOI:** 10.1371/journal.pone.0051514

**Published:** 2012-12-12

**Authors:** Lina Qiao, Liang Xie, Kun Shi, Tongfu Zhou, Yimin Hua, Hanmin Liu

**Affiliations:** 1 Department of Pediatric Cardiology, West China Second University Hospital, Sichuan University, Chengdu, China; 2 Key Laboratory of Obstetric & Gynecologic and Pediatric Diseases and Birth Defects of Ministry of Education, West China Second University Hospital, Sichuan University, Chengdu, China; 3 Department of Cardiology, Chengdu Women & Children Center Hospital, Chengdu, China; Vanderbilt University Medical Center, United States of America

## Abstract

Pulmonary hypertension (PH) is a fatal disease that lacks an effective therapy. Notch signaling pathway plays a crucial role in the angiogenesis and vascular remodeling. However, its roles in vascular remodeling in PH have not been well studied. In the current study, using hypoxia-induced PH model in rat, we examined the expression of Notch and its downstream factors. Then, we used vessel strip culture system and γ-secretase inhibitor DAPT, a Notch signaling inhibitor to determine the effect of Notch signaling in vascular remodeling and its potential therapeutic value. Our results indicated that Notch 1–4 were detected in the lung tissue with variable levels in different cell types such as smooth muscle cells and endothelial cells of pulmonary artery, bronchia, and alveoli. In addition, following the PH induction, all of Notch1, Notch3, Notch4 receptor, and downstream factor, HERP1 in pulmonary arteries, mRNA expressions were increased with a peak at 1–2 weeks. Furthermore, the vessel wall thickness from rats with hypoxia treatment increased after cultured for 8 days, which could be decreased approximately 30% by DAPT, accompanied with significant increase of expression level of apoptotic factors (caspase-3 and Bax) and transformation of vascular smooth muscle cell (VSMC) phenotype from synthetic towards contractile. In conclusion, the current study suggested Notch pathway plays an important role in pulmonary vascular remodeling in PH and targeting Notch signaling pathway could be a valuable approach to design new therapy for PH.

## Introduction

Pulmonary hypertension (PH) is a fatal clinical diseases that can be idiopathic or secondary to heart, pulmonary, and vascular diseases. Importantly, PH is one of the important factors determining the prognosis of patients with congenital heart defects. Currently, there is no effective treatment for severe PH patients, who usually have low quality of life and poor prognosis. Pulmonary vascular remodeling is a common feature of severe PH regardless of primary cause, suggesting that intervention of pulmonary vascular remodeling may be valuable for pulmonary hypertension, which was supported by animal studies and evidence-based studies [1], [2].

The most important characteristic of pulmonary vascular remodeling is the thickening of the vascular intima, media, and adventitia, which is generally thought to result from cell hypertrophy, proliferation, migration, and extracellular matrix deposition. This process involves many cell types, including endothelial cells, smooth muscle cells, fibroblasts, inflammatory cells, and platelets. These cells produce and respond to a variety of factors, such as endothelin, angiotensin II, transforming growth factor beta (TGF-β), platelet-derived growth factor (PDGF), fibroblast growth factor (FGF ), vascular endothelial growth factor (VEGF), serotonin, and angiopoietin as well as intracellular signaling molecules such as tyrosine kinases, mitogen-activated protein kinases (MAPKs), protein kinase C (PKC), and phosphatidylinositol 3-kinase (PI3K), Rho kinases, SMAD and calcium ion channels [3], [4], . However, because of the complicated process of PH, the potentially effective molecular targets remain to be defined [1], [2], [3], [4], [5], [6], [7]. In the past decades, extensive investigation have been conducted to study whether angiogensis and vascular remodeling factors involved in the development of mammal embryos could be targeted for the intervention of PH. For example, VEGF, TGF-β, PDGF, Angiopoietin/Tie, FGF, and Ephrin/Eph signal pathways have been determined and reported in the literature [8], [9], [10], [11], [12]. One of the major finding is that Notch signaling pathway plays a crucial role in the angiogensis and vascular remodeling [8], [9], [10], [11], [12], [13], [14], [15], [16], [17], [18].

The Notch system is highly conservative in evolution and plays an important role in cell proliferation, differentiation, and apoptosis. To date, four Notch receptors (Notch1, Notch 2, Notch 3, and Notch 4), and five ligands (Jagged1, Jagged 2, Delta-like 1, Delta-like 2, and Delta-like 3) have been determined [19], [20], [21]. Owing to the transmembrane domain of the Notch ligands and receptors, the signal transduction of Notch system is primarily based on cell-to-cell contact [19], [20], [21], [22]. It has been found that Notch receptors 1, 3 and 4 and ligands Dll4, Jagged1 and 2 are mainly expressed in the arterial system in human and critical for maintenance of normal vascular structure, angiogenesis, and vascular remodeling in both physiological and pathological conditions [9], [10], [13], [16], [17], [18]. The indispensable roles of Notch system have been supported by the fetal lethality of Notch signaling deficiency. For example, either knockout of Notch1, Notch1 plus Notch4, HERP1 plus HERP2, Jagged1, DLL4, presenillin-1, or constant expression of Notch4 results in embryonic death due to vascular remodeling defects [9], [10], [13], [16], [17], [20], [23]. In addition, gene mutation of Notch system has been observed in human diseases with involvement of artery, such as Algille Syndrome, which is associated with Jagged1 gene mutations and narrowing of small pulmonary arteries, as well as CADASIL Syndrome, which results from mutations of Notch3 gene and progressive degeneration of the vascular smooth muscle cells (VSMCs) [9], [10], [13], [16], [17], [20]. However, its role in vascular remodeling in PH has not been well elucidated. To date, there were about three studies focusing on the effect of Notch signaling pathway in vascular remodeling of PH. [24], [25], [26] Li etc found that overexpression of NOTCH3 existed in the lungs of humans and rodents with pulmonary hypertension, knock-out mice with homozygous deletion of Notch3 did not develop pulmonary hypertension in response to hypoxic stimulation and pulmonary hypertension could be successfully treated in mice by administration of N-[N-(3,5-difluorophenacetyl)-L-alanyl]-S-phenylglycine t-butyl ester (DAPT), a γ-secretase inhibitor [24]. Also, our previous work found that DAPT could inhibit pulmonary vascular remodeling induced by angiotensin II and PDGF ex vivo [25], [26]. However, Notch signaling pathway is very complicated. The mechanism of the effects of this pathway on PH is still unclear. In this study, we examined the expression of Notch 1–4 receptors and downstream factors during the development of PH and tried to determine the role of Notch signaling in vascular remodeling of PH by using DAPT as an inhibitor of Notch signaling pathway.

## Materials and Methods

### Ethical Statements

All these cleaning laboratory Wistar rats were obtained from Experimental Animal Center of West China Medical Center in Sichuan University. The Committee on the Ethics of Animal Experiments of Sichuan University approved all protocols related to the animal experiments in this study and the study was carried out in accordance with the National Institute of Health guideline for the Care and Use of Laboratory Animals.

### Hypoxia Experiments

Thirty-five adult Wistar rats (average age: 8 weeks; weight: 200–220 g) were divided into seven groups and received hypoxia treatment for designated duration of time: 0, 1, and 3 days, or 1, 2, 3 and 4 week, respectively. Accordingly, the experimental groups were named as control, hypoxia-one-day, hypoxia-three-day, hypoxia-one-week, hypoxia-two-week, hypoxia-three-week, hypoxia-four-week.

For one-day treatment, rats stayed in a hypoxia chamber with 10%±1% oxygen, less than 3% carbon dioxide, and normal atmospheric pressure for 8 hours everyday. The concentrations of oxygen and carbon dioxide were monitored every 10–15 minutes. Food and water were provided normally. The control rats were kept in the same conditions except hypoxia. The remaining treatment regiments are the expansion of this one-day treatment according to the designated time duration. Following designated hypoxia treatment, rats were anaesthetized at 0.5–1 hour after leaving the hypoxia chamber by 10% chloral hydrate (400 mg/kg, i.p). The pressures were measured in right ventricle and pulmonary artery via right external jugular vein intubation. The heart and lungs were isolated after cervical dislocation. Fulton index was calculated as RV/(LV+S). The left lungs were dissected for isolating pulmonary arteries: fixing the left pulmonary artery by a pair of forceps and dissecting the lung tissue as far as possible by ophthalmic scissors. These pulmonary arteries were kept in liquid nitrogen immediately for mRNA extraction. And the right lungs were fixed in formalin and embedded in paraffin for Hematoxylin-eosin (HE) and immunohistochemistry (IHC) staining.

### Pulmonary Artery Vascular Strips Culture

24 pulmonary artery strips were collected from rats following hypoxia treatment for 28 days by isolating and cutting open the pulmonary arteries via vertical axis. 700 µl rat rail collagen, 100 µl M199, 200 µl sterile NaHCO_3_ (11.76 mg/ml) were mixed on ice and spread in 35 mm petri dishes. The strips were put in the dishes with endothelium on the top and incubated at 37°C with 5% CO2 for 15 minutes. Then, 1.5 ml M199 medium with 5% FBS and 100 U/ml streptomycin and penicillin were added to the dishes and dish was returned to the incubator. The culture medium was replaced with fresh medium every day for seven days. Each time the renewed culture medium was added by 3 µl M199, 3 µl DMSO, 3 µl 0.5 mmol/l DAPT (N-[N-(3,5-Difluorophenacetyl)-L-alaryl]-S-phenylalycine t-butyl ester, C23H26F2N2O4, Sigma D5942), and 3 µl 5 mmol/l DAPT respectively according to the different experimental groups. On the ninth day of the culture, the strips were cut into two parts, one part was kept in liquid nitrogen for extraction of mRNA and the other was fixed in formalin and embedded in paraffin for HE and IHC staining.

### RT-PCR

Total RNA was isolated using Trizol (MRC, U.S.A). cDNA was synthesized by the RevertAid™ First Strand cDNA Synthesis Kit (MBI, Lithuania). Real-time PCR was performed by SYBR Green I (Roche, Switzerland) method. The reaction included cDNA, primers (table 1, Takara, China), dNTP (Promega, U.S.A), and Taq DNA polymerase (BioDev, China). The PCR procedure was as follows: pre-denaturing at 94°C for 2 minutes, and 45 cycles of denaturing at 94°C for 20 seconds, annealing at 52°C for 30 seconds, elongation at 72°C for 40 seconds.2^−ΔΔCt^ method was applied for relative quantification of the target genes. The 2^−ΔΔCt^ value of RT-PCR was in a skew distribution and were logarithmically transformed for further analyses.

**Table 1:** The sequences of primers used in this study.

*Gene Name*	*Primers*	*Product Size (bp)*
Notch1	Forward: 5′-TCTCAACTGCCAGAACCTTGT-3′ Reverse: 5′-ATGCCTCGCTTCTGTGCAG-3′	171
Notch2	Forward: 5′-CACTGAGAGCTCCTGTTTCAA-3′ Reverse: 5′-CAGGCCATCAACACACGTTC-3′	160
Notch3	Forward: 5′-ATGGCAGGCTTCACAGGAAC-3′ Reverse: 5′-TGCAGCTGAAGCCATTGACT-3′	109
Notch4	Forward: 5′-AGTGTCTCCCAGGCTTTGAA-3′ Reverse: 5′-GAAGATCAAGGCAGCTGGCT-3′	96
HERP1	Forward: 5′-GATGCTCCAGGCAACAGG-3′ Reverse: 5′-GTGGGTCCGAAGGGTCAA-3′	143
HERP2	Forward: 5′-CCAACCACATCGTCCCA-3′ Reverse: 5′-CTAGCTTCGCAGATCCCTG-3′	145
SM-MHC	Forward: 5′-ATGCTGGGAAGGTGGACTAT-3′ Reverse: 5′-CGGTCCACATCCTTCCACA-3′	133
SM22α	Forward: 5′-TCTGAGCAAGTTGGTGAACA-3′ Reverse: 5′-GAGGTCAACAGTCTGGAACAT-3′	169
MGP	Forward: 5′-GCCCTGTGCTATGAATCTCA-3′ Reverse: 5′-GCGTGCCATCTCTGCTGA-3′	107
OPN	Forward: 5′-CACTCAGATGCTGTAGCCACTT-3′ Reverse: 5′-GTTGCTTGGAAGAGTTTCTTGCT-3′	126
Bcl-Xl	Forward: 5′-CCTTCATAAGAGCCACAAAGA-3′ Reverse: 5′-ATGGGCTCAACCAGTCCATT-3′	130
BAX	Forward: 5′-GTTTCATCCAGGATCGAGCA-3′ Reverse: 5′-CAATCATCCTCTGCAGCTCC-3′	156
GAPDH	Forward: 5′-CCTCAAGATTGTCAGCAAT-3′ Reverse: 5′-CCATCCACAGTCTTCTGAGT-3′	141

### Measurement of the Thickness of Vessel Wall

The thickness of vessel wall in strips was measured by Verhoeff iron hematocylin stain method. The elastic fiber was black or dark blue after staining. The results were scanned and analyzed using Image-Pro Plus software. Four fields were selected randomly for one strip. The distance between inner and outer elastic fibers was measured for three times in one field. The average of the twelve distance values was used as the thickness of vessel wall in this strip.

### Immunohistochemistry

The IHC was conducted following the manufacture instruction for kits SP-9001, SP-9002, and SP-9003 (Zhong Shan -Golden Bridge Biological Technology CO., LTD, Beijing). The following antibodies were used: Notch1 (sc-6014, Santa Cruz Biotechnology, Inc.), Notch2 (sc-7423, Santa Cruz Biotechnology, Inc.), Notch3 (sc-7424, Santa Cruz Biotechnology, Inc.), Notch4 (sc-8646, Santa Cruz Biotechnology, Inc.), PCNA (Neomarker, MS-106-P, Lab Vision Co.), and Caspase-3 (Neomarker, MS-1121-P, Lab Vision Co.). PCNA and caspase-3 positive cells were counted from five random fields (×400) of one strip. The positive rate was calculated by dividing the number of all positive cells by the total number of cells in the five fields.

### Statistics

The SPSS program software (version 13.0, SPSS Inc., USA) was used for statistical analysis. Quantitative data were expressed as mean ± s.e.m. One-way or repeated-measure ANOVA. was used to analyze the difference among groups. LSD for homogeneity of variances, or Tamhane’s T2 for heterogeneity of variances was used for the multiple comparisons of the means of each group. The differences were considered significant when p<0.05.

## Results

### Validation of Rat PH Model Induced by Hypoxia

**Figure 1:**
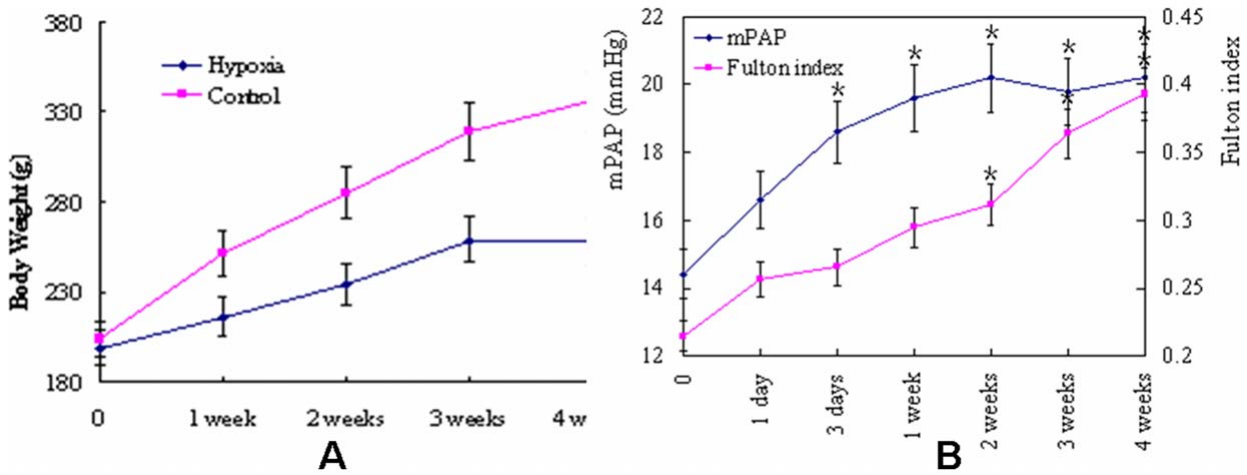
Validation of rat PH model induced by hypoxia. (A) weight changes in the course of hypoxia treatment. The results of F test showed that there were significant differences between the weights of experimental and control groups at each time points. The curve of weight changes in control rats is nearly a straight line, whereas the curve in model rats is nearly a quadratic function curve. (B) the changes of mean pulmonary artery pressures (mPAP) and the Fulton index in PH rats. ANOVA analyses revealed that mPAP and the Fulton index were significantly different in each points. With the increase of hypoxia duration, the mPAP in hypoxia rats increased with a peak at the second week, and maintained a high level until the end point. The continuous increase of Fulton index suggested progressive hypertrophy of rat right ventricle. (*, P<0.05 compared to control group, Data are expressed as means ± s.e.m.)

**Figure 2:**
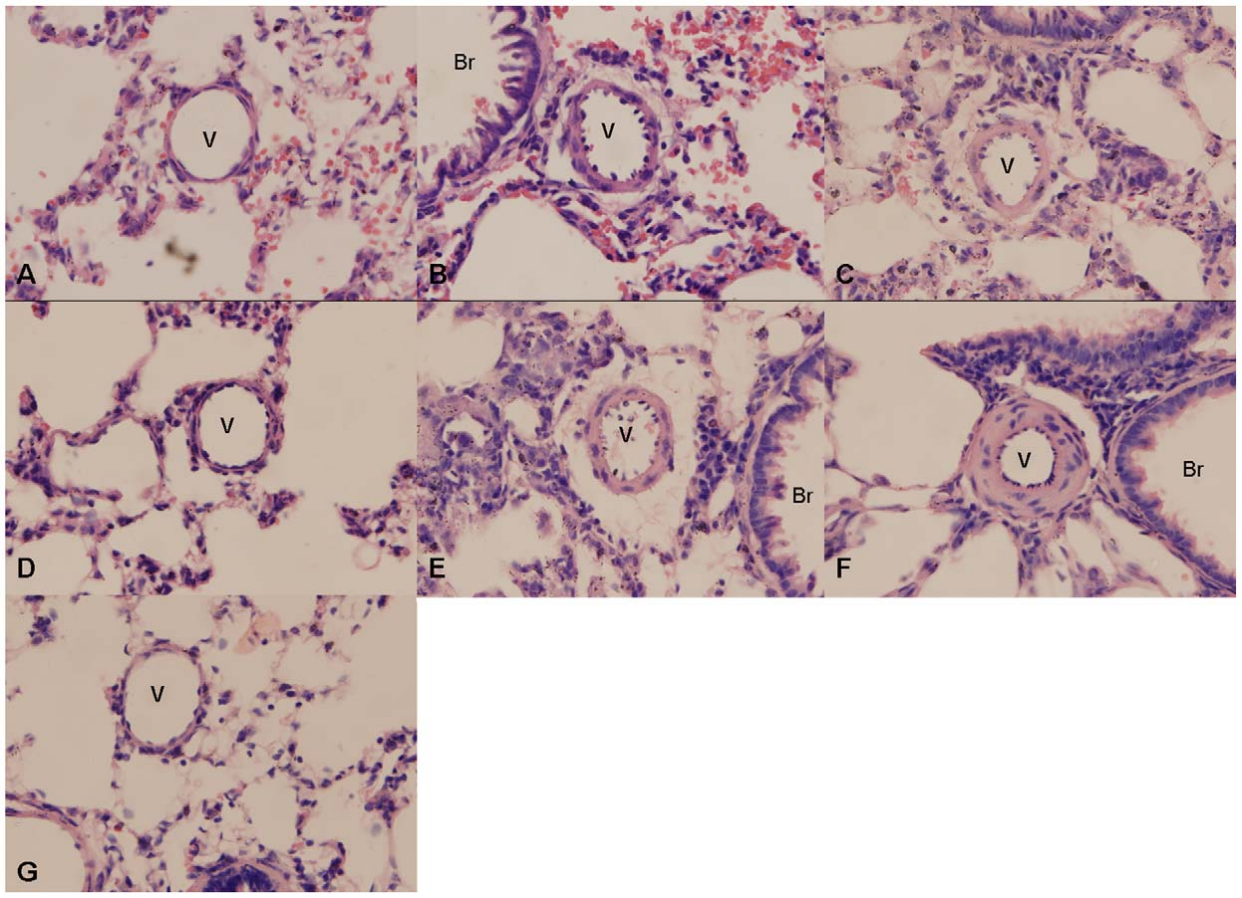
Hematoxylin-eosin staining of rat lung tissue. (A–G) HE staining of lung tissues of hypoxia rats for 1, 3 days, and 1, 2, 3, 4 weeks, and normal rats, respectively. (×400) V: Vessel; Br: Bronchium.

Body weight, mean pulmonary artery pressure (mPAP) and the Fulton index of rat have been obtained to validate the successful induction of PH. As shown in Figure 1A, the body weight of hypoxia rats is significantly lower than that of normoxia rats (p<0.001). The curve of weight changes in control rats was nearly a straight line, whereas the curve in model rats was nearly a quadratic function curve. The changes of the mPAP and the Fulton index of the hypoxia rats are shown in Figure 1B. The mPAP increased and reached the peak at the second week since the initiation of hypoxia treatment. The Fulton index progressively increased, suggesting the right ventricle thickening continuously progressed. The HE staining for lung tissues are shown in Figure 2. The increased thickness of pulmonary arterioles in the hypoxia rats indicated the progressive worsening of pulmonary vascular remodeling. Collectively, it was suggested that PH has been successfully induced with the hypoxia treatment.

### The Gene Expression of Notch Receptors and their Downstream Effectors in PH Rats

**Figure 3:**
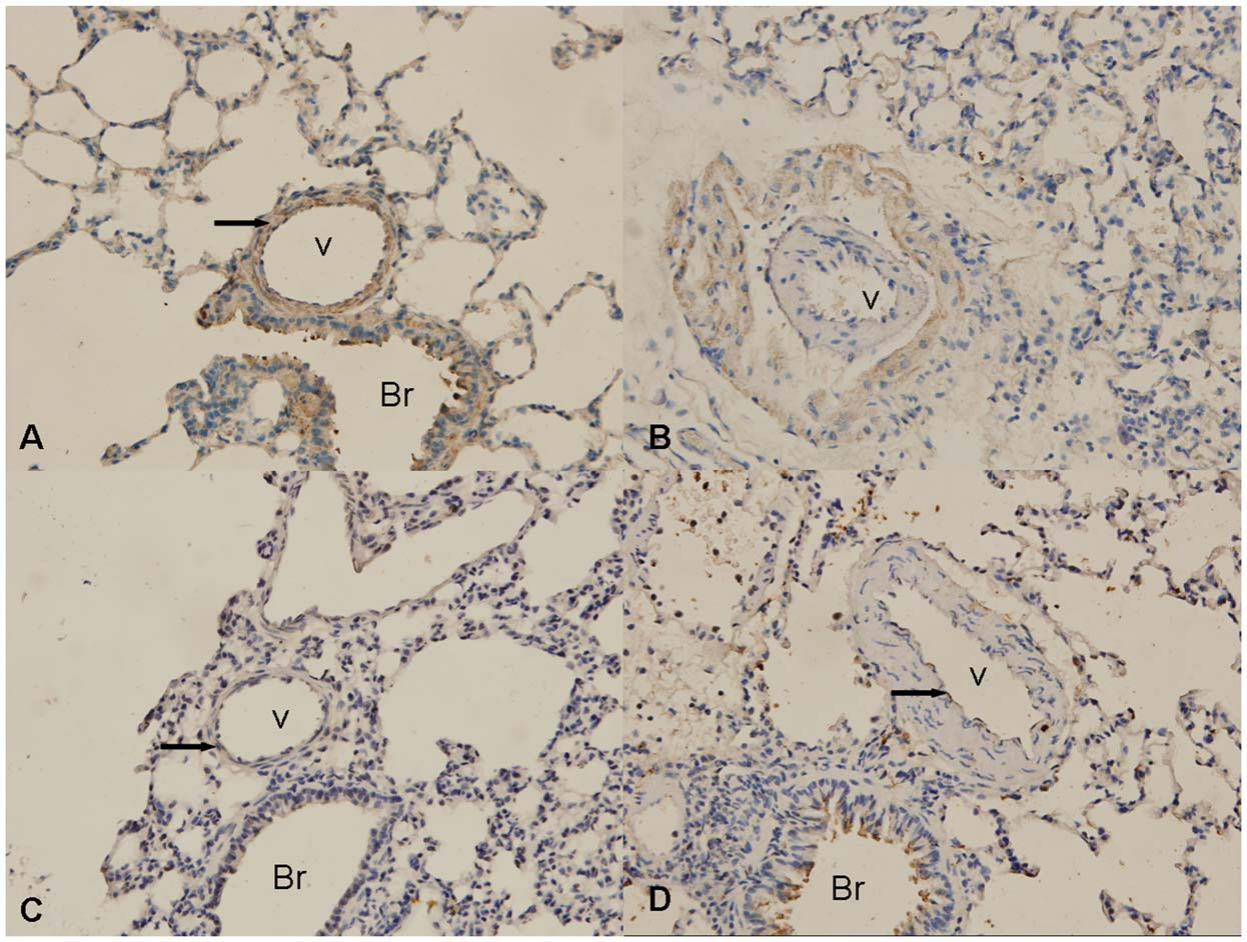
Immunohistochemistry staining of Notch1-4 receptors in rat lung tissues. (A–D) immunohistochemistry staining of Notch1-4 receptors in pulmonary artery of normal rats, respectively. (×400) V: Vessel; Br: Bronchium; Arrow: positive staining.

Next, using IHC, we examined Notch receptor expression following the induction of PH. Our results revealed that the positions where each of these four Notch receptors located in PH rats were the same as the positions in normal rats. Notch1 located in cell membrane and cytoplasm, Notch 2 and Notch 4 located in cell membrane, whereas Notch 3 located in nucleus in rat lung tissues (Figure 3). In addition, Notch1 was detected in smooth muscle cells and endothelial cells of pulmonary artery, smooth muscle cells, epithelial cells, and macrophages of bronchia. Notch2 expressed in epithelial cells of bronchia and alveoli. Notch3 was observed in smooth muscle cells of pulmonary artery and epithelial cells of bronchia. Notch4 was present in endothelial cells of pulmonary artery and epithelial cells of bronchia, little in smooth muscle cells of pulmonary artery.

**Figure 4:**
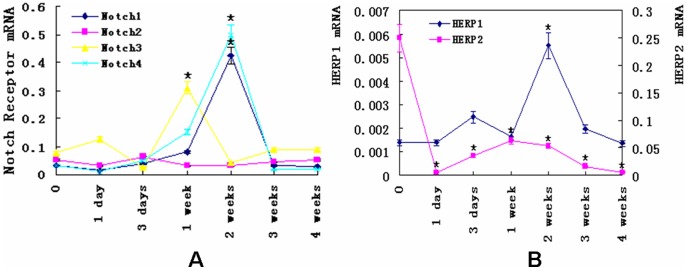
The gene expression of Notch receptors and their downstream effectors in PH rats. (A) the change of mRNA levels of Notch1-4 receptors in lung tissues of hypoxia rats. Notch3 mRNA level reached the peak at 1 week, whereas Notch1 and Notch4 did at 2 weeks. Compared to the baseline, the increase of Notch3 mRNA level was two folds, Notch1 four folds, and Notch4 five folds. All of them returned to the baseline by 3 weeks. (B) the change of mRNA levels of HERP1 and HERP2 in lung tissues of hypoxia rats. The mRNA level of HERP1 reached the peak at the second week and returned to basa level at the following days. The mRNA level of HERP2 decreased at the first day and maintained a low level in the following days. (*, P<0.05 compared to control group, Data were normalized to GAPDH and expressed as means ± s.e.m.)

Regarding the change of mRNA levels, Notch1, Notch3, Notch4 receptor expression exhibited different patterns along the course of hypoxia treatments (Figure 4A). The peak of Notch3 was 1 week, and 2 weeks for Notch1 and Notch4. Compared to the control, Notch3 mRNA level was increased two folds, 4 folds for Notch1, and 5 folds for Notch4. Then they returned to the baseline level. The gene expression changes of Notch downstream genes, HERP1 and HERP2, were also changed (Figure 4B). While HERP1 reached the peak at the second week and gradually dropped to the base level, HERP2 declined quickly at the first day of hypoxia and maintained at the low level.

### The Wall Thickness Changes of Vascular Strip in Culture

**Figure 5:**
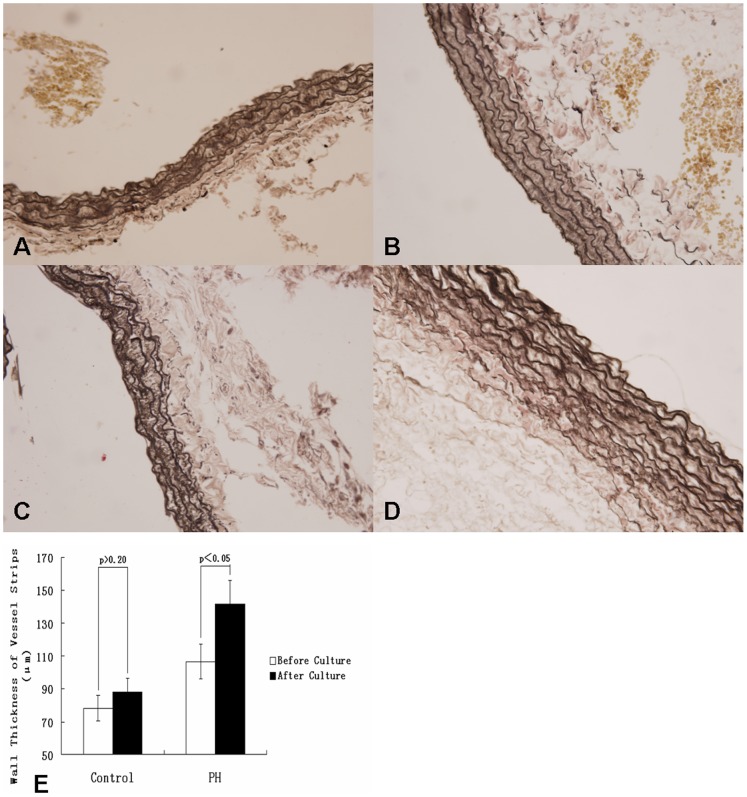
The wall thickness changes of vascular strips in culture. (A–D) Verhoeff iron hematocylin staining of Pulmonary artery vascular strips (×400): (A) pulmonary artery of control rat; (B) pulmonary artery of rat with 4-week-hypoxia; (C) pulmonary artery of control rat cultured for 8 days; (D) pulmonary artery of rat with 4-week-hypoxia cultured for 8 days. (E) the change of wall thickness of vessel strips in control and PH groups. Compared to the control, the thickness of vascular media of the hypoxia rats was significantly increased. While there was no significant difference between the strips of the control rats before and after culturing (p>0.20), the wall thickness of the strips from the hypoxia rats after culturing were significantly thicker than those before culturing, increasing about 30 µm. (*, P<0.05 compared to control group. Data are expressed as means ± s.e.m.)

In the extended culture study, we measured the changes of vascular strip wall thickness in culture. As shown in Figure 5, the thickness of vascular wall of the strips of four groups were different significantly (p<0.05). The thickness of vascular media of the hypoxia rats increased significantly, it was about 30%, when PH occurred. Of note, there was no significant difference between the strips of the normoxia rats before and after culturing (p>0.20). In contrast, the strips of the hypoxia-treated rats were significantly thicker after culturing than before, increasing about 30 µm, nearly 30%. It was suggested that vascular strips from PH rats have a prolonged proliferative trait in culture.

### The Impact of DAPT on Notch Signaling in Vascular Strips from PH Rats

**Figure 6:**
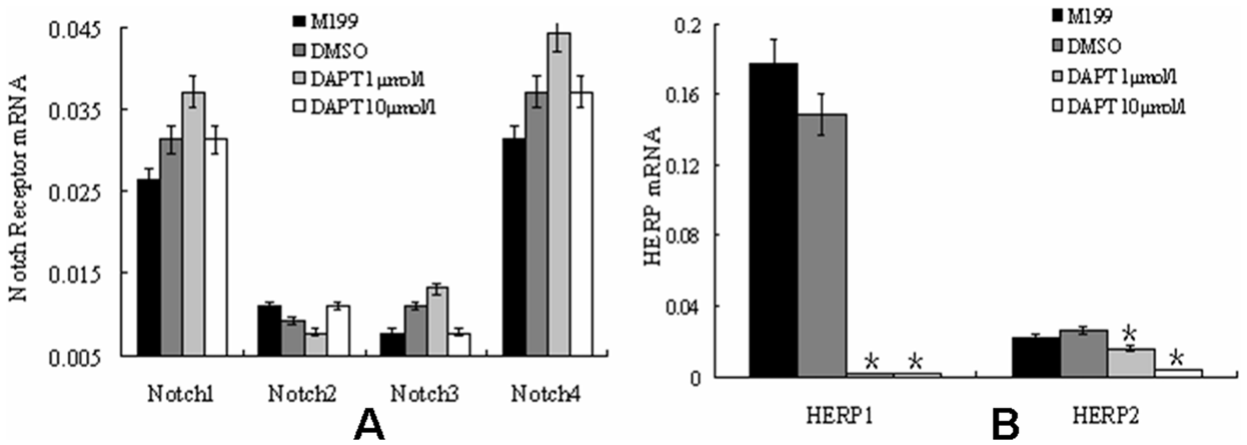
The impact of DAPT on Notch signaling in vascular strips from PH rats. (A) the effects of DAPT on the mRNA level of Notch receptors in vessel strips of hypoxia rats. No significant differences of Notch receptors mRNA levels among experimental groups were detected by ANOVA. (B) the effects of DAPT on the mRNA levels of HERP1 and HERP2 in vessel strips of hypoxia rats. The mRNA levels of HERP1 and HERP2 were significantly lower In both low and high dosage of DAPT groups than in blank and DMSO control groups. However, there were no significant differences between blank and DMSO control groups, or between DAPT-low and DAPT-high groups. (*, P<0.05 compared to control group. Data were normalized to GAPDH and expressed as means ± s.e.m.)

In the classic Notch signal transduction, following the binding of ligands to Notch receptors, two steps of enzymolysis occurs. As the result, the Notch intracellular domain (NICD) will be released, which then translocate to nucleus and activate the Notch signaling. The second enzymolysis is mediated by presenilin-dependent γ-secretase. DAPT is a specific inhibitor of γ-secretase that inhibits the hydrolysis of Notch protein. Accordingly, we determined whether the administration of DAPT influence the expression of Notch system. It was found that none of 4 Notch receptor mRNA levels showed significant differences following DAPT treatment when compared to the control (Figure 6A). However, the expressions of HERP-1 and HERP-2 genes were significantly inhibited in DAPT-low and DAPT-high groups, comparing with blank and DMSO control groups (Figure 6B, p<0.05).

### DAPT Inhibits the Increase of Wall Thickness in Cultured Vascular Strips from PH Rats

**Figure 7:**
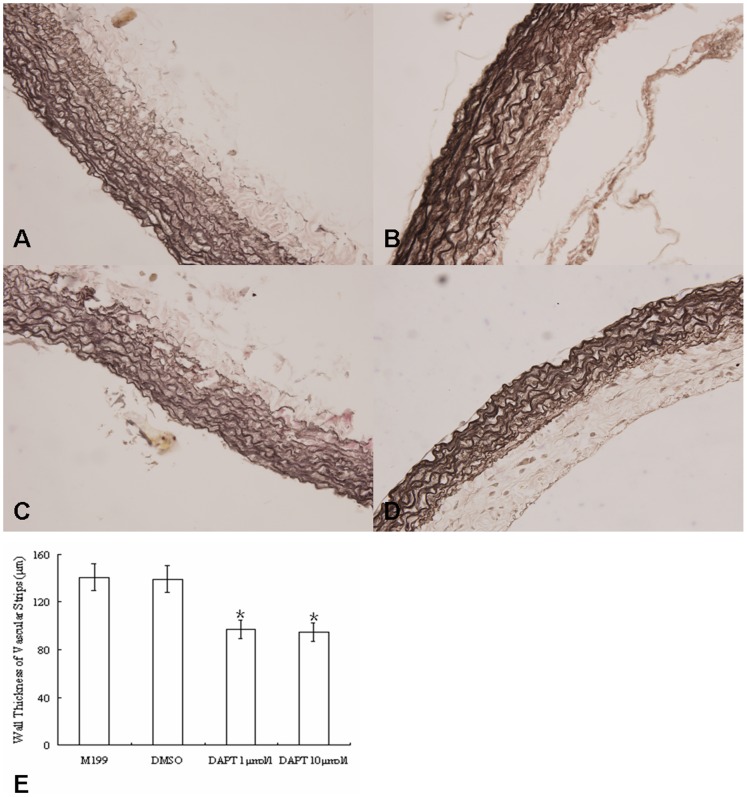
DAPT inhibits the increase of wall thickness in cultured vascular strips from PH rats. (A–D) Verhoeff iron hematocylin staining of Pulmonary artery vascular strips (×400): (A) pulmonary artery in culturing lung tissue strip treated with additional equivalent M199; (B) pulmonary artery in culturing lung tissue strip treated with DMSO; (C) pulmonary artery in culturing lung tissue strip treated with DAPT (1 µmol/l); (D) pulmonary artery in culturing lung tissue strip treated with DAPT (10 µmol/l). (E) the change of vascular media thickness of hypoxia rats with or without DAPT treating. The vascular media of hypoxia rats treated with DAPT were significantly thinner than those treated with M199 or DMSO. (*, P<0.05 compared to control group. Data are expressed as means ± s.e.m.)

Next, we determined the thickness of vascular wall in presence of DAPT in the culture. As shown in Figures 5&7, in the absence of DAPT, the thickness of vascular wall in rats of hypoxia treatment increased significantly (p<0.05). However, DAPT treatment decreased the thickness of vascular strips when compared to blank and DMSO controls.

**Figure 8:**
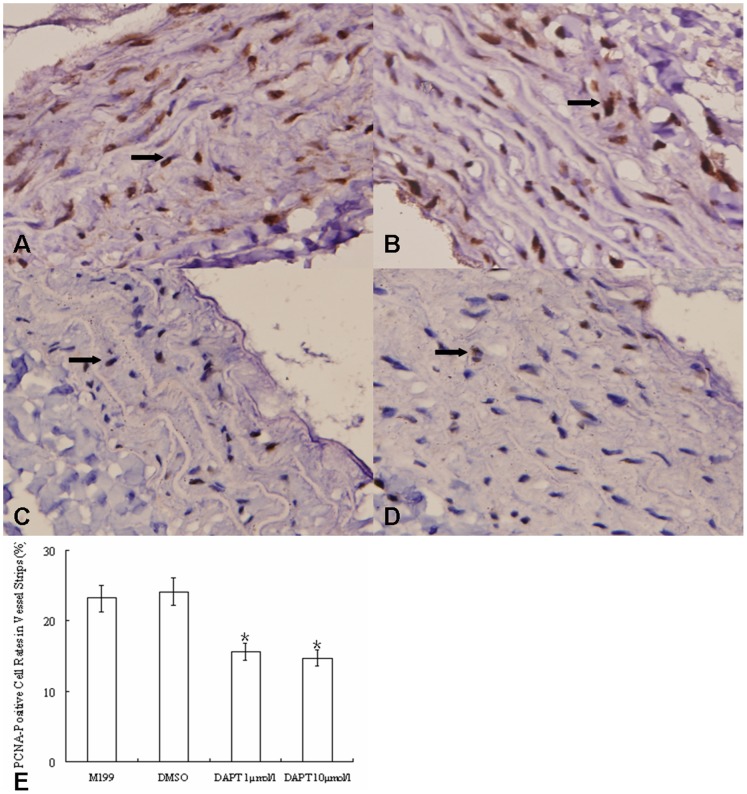
DAPT inhibits the proliferation of cultured vascular strips from PH rats. (A–D) PCNA immunohistochemistry staining in pulmonary artery strips (×400, nuclear staining): (A) pulmonary artery in culturing lung tissue strip treated with additional equivalent M199; (B) pulmonary artery in culturing lung tissue strip treated with DMSO; (C) pulmonary artery in culturing lung tissue strip treated with DAPT (1 µmol/l); (D) pulmonary artery in culturing lung tissue strip treated with DAPT (10 µmol/l). (E) the change of PCNA-positive cell rate in vessel strips from hypoxia rats with or without DAPT treatment. The PCNA-positive cell rates in vessel strips from hypoxia rats treated with DAPT were significantly lower than those of blank and DMSO control hypoxia rats. However, the PCNA-positive cell rates between the two DAPT treating groups or between the blank and DMSO control groups were not significantly different. (*, P<0.05 compared to control group. Data are expressed as means ± s.e.m.) Arrow: positive staining.

PCNA is a co-factor of DNA polymerase δ involved in DNA synthesis and associated with cell proliferation. To understand the DAPT-mediated inhibition of vascular wall thickening, we determined the presence status of PCNA among different culture conditions. It was found that PCNA positive rates in blank control and DMSO control groups were higher than in DAPT-low and DAPT-high groups. There were no significant differences between blank and DMSO control groups, or between DAPT-low and DAPT-high groups (Figure 8), suggesting DAPT mediated an effect of anti-proliferation.

### The Effects of DAPT on the Phenotype of Vascular Smooth Muscle Cells (VSMCs) in Culturing Strips from PH Rats

**Figure 9:**
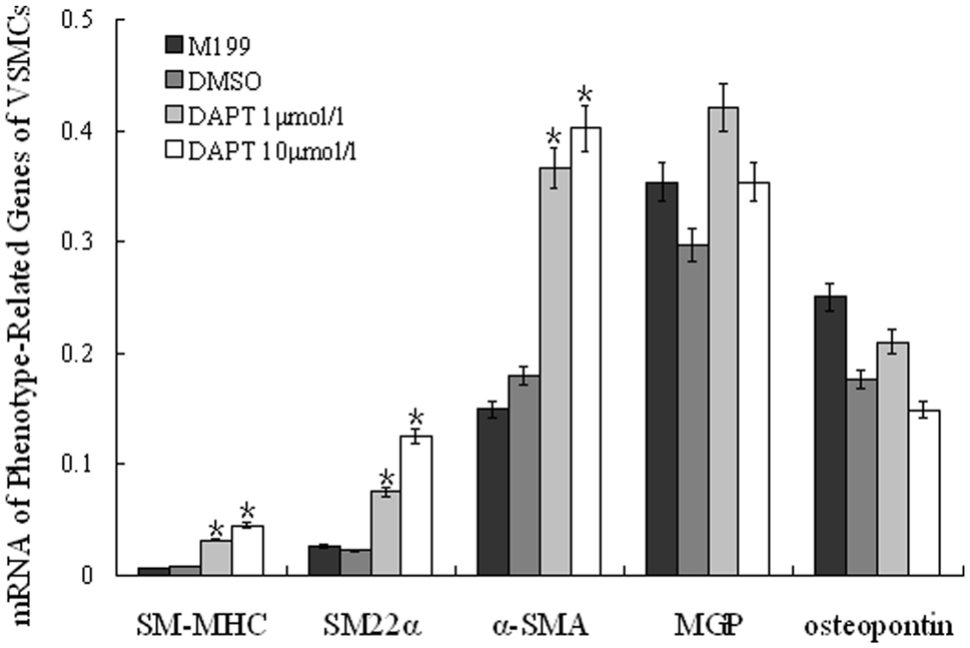
The effect of DAPT on the expression of phenotype-related genes of VSMCs from hypoxia rats. Compared to blank and DMSO control groups, the expressions of SM-MHC and SM22α in both low- and high- dosage of DAPT groups were significantly increased (p<0.05). There were no significant differences of expression of these two genes between low- and high- dosage of DAPT groups, or between blank and DMSO control groups, respectively (p>0.2). The expressions of MGP and osteopontin were not significantly different in the four groups (p>0.2). (*, P<0.05 compared to control group. Data were normalized to GAPDH and expressed as means ± s.e.m.)

According to the structure and function difference, two phenotypes of VSMCs have been defined: contractile and synthetic. The contractile VSMCs are full of muscle fibers with less cell organelles such as rough endoplasmic reticulum and Golgi apparatus, and express some specific proteins such as SM-MHC and SM22α, and the former is the most reliable marker of the mature contractile VSMCs [27]. The synthetic VSMCs are lack of muscle fibers but contain rich cell organelles such as rough endoplasmic reticulum, ribosome, and Golgi apparatus, Synthetic VSMCs proliferate actively, and synthesize matrix proteins, such as matrix Gla protein (MGP) and osteopontin which are considered as the specific markers of the synthetic VSMCs [28]. In the current study, to determine the impact of Notch signaling on VSMC phenotypes, we measured the mRNA levels of SM-MHC, SM22α, MGP, and osteopontin in cultured strips in the absence or presence of DAPT. Our results revealed that the mRNA levels of SM-MHC and SM22α were significantly lower in blank and DMSO control groups than in DAPT-low and DAPT-high groups (p<0.05). There were no significant differences of the mRNA levels of SM-MHC and SM22α either between blank control and DMSO control groups, or between DAPT-low and DAPT-high groups. Furthermore, there were no significant differences of the mRNA levels of MGP and osteopontin among blank control, DMSO control, DAPT-low, and DAPT-high groups (Figure 9). These results suggested that inhibition of Notch signaling by DAPT, promoted the expression of contract proteins in pulmonary arterial smooth muscle cells and facilitated the development of contractile phenotype.

### The Effects of DAPT on Apoptosis of VSMCs in Culturing Strips from PH Rats

**Figure 10:**
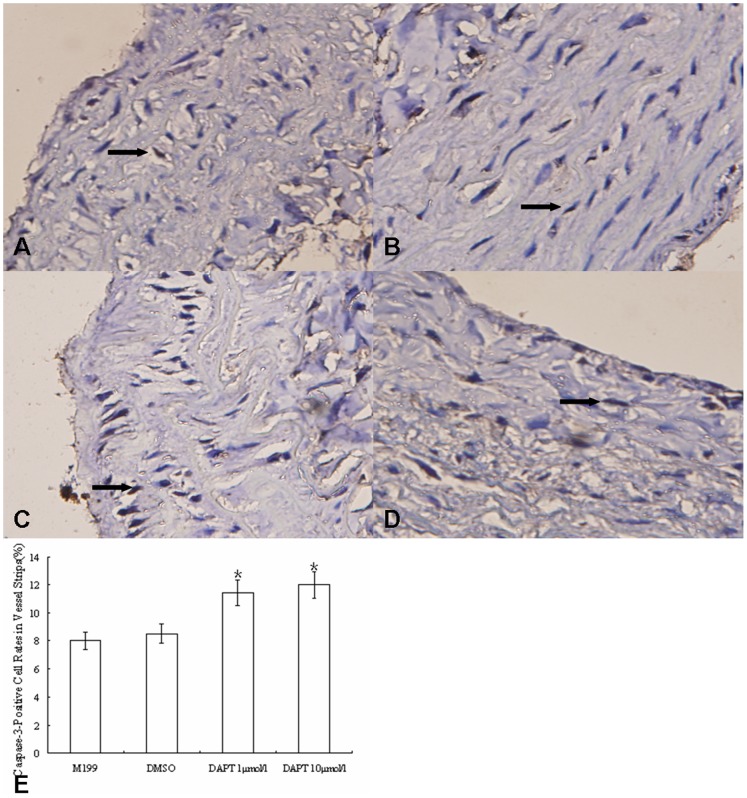
The change of caspase-3-positive rates of vascular wall cells from hypoxia rats with or without DAPT treating. (A–D) Caspase-3 immunohistochemistry staining in pulmonary artery strips (×400, cytoplasmic staining): (A) pulmonary artery in culturing lung tissue strip treated with additional equivalent M199; (B) pulmonary artery in culturing lung tissue strip treated with DMSO; (C) pulmonary artery in culturing lung tissue strip treated with DAPT (1 µmol/l); (D) pulmonary artery in culturing lung tissue strip treated with DAPT (10 µmol/l). (E) The change of caspase-3-positive rate of vascular wall cells from hypoxia rats with or without DAPT treating. The caspase-3-positive cell rates in vessel strips from hypoxia rats treated with DAPT (1 µmol/l) or (10 µmol/l) were significantly higher than those treated with M199 (blank control)or DMSO(p<0.05). However, the caspase-3-positive cell rates between the two DAPT treatment groups or between the blank and DMSO control groups were not significantly different. (*, P<0.05 compared to control group. Data are expressed as means ± s.e.m.) Arrow: positive staining.

Caspase-3 is an effect factor in caspase-mediated apoptosis, which plays an indispensable role in apoptosis. The expression of caspase-3 is widely used for estimation of apoptosis. Therefore, the percentages of caspase-3 positive cells were measured in the present study and they were significantly higher in DAPT-low and DAPT-high groups than blank control and DMSO control groups. There were no significant differences between blank control and DMSO control groups, or between DAPT-low and DAPT-high groups. (Figure 10).

**Figure 11:**
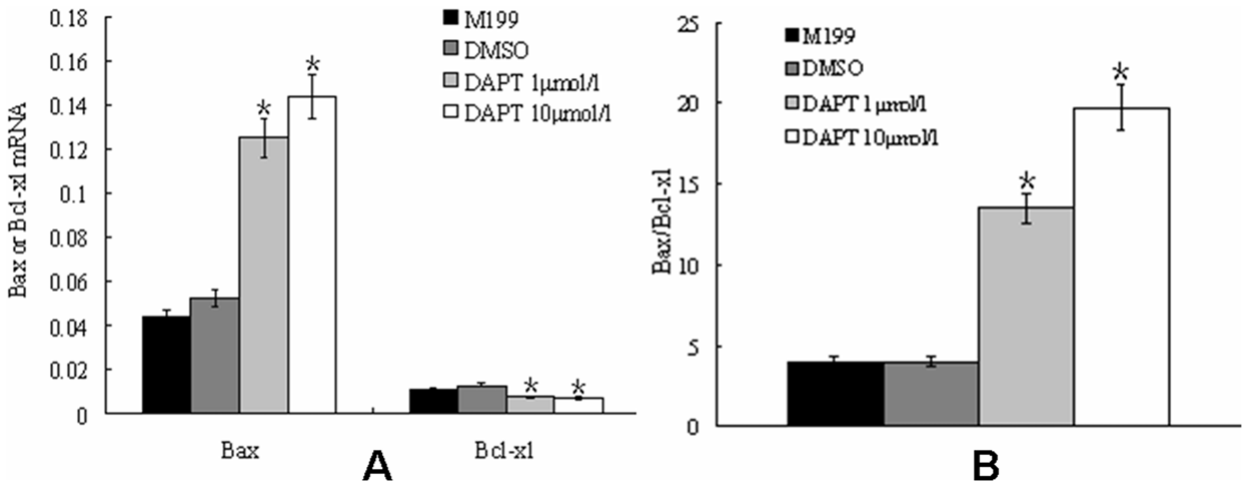
The effect of DAPT on Bax and Bcl-xl mRNA expressions in pulmonary artery strips from hypoxia rats. The mRNA level of Bax increased while the mRNA level of Bcl-xl decreased after DAPT treatment. The ratio of Bax mRNA to Bcl-xl mRNA significantly increased after DAPT treating. (*, P<0.05 compared to control group. Data were normalized to GAPDH and expressed as means ± s.e.m.)

Bax promotes apoptosis, whereas Bcl-xl promotes cell survival. When the ratio of Bax to Bcl-xl is high, the cell is more prone to apoptosis, or vice versa. Our results indicated that DAPT treatment led to an increase of Bax mRNA level, and decrease of Bcl-xl level in vascular strips, resulting in a significant increase of Bax/Bcl-xl ratio and apoptosis (Figure 11). Collectively, we postulated that DAPT may attenuate PH by inhibiting proliferation and inducing apoptosis through regulating caspase-3, Bax, and Bcl-xl expression.

As to the culturing strips from normal rats, DAPT could also impact on Notch signaling (Figure S1). However, DAPT did not effect on the wall thickness (Figure S2). Meanwhile, there were no significant changes of PCNA positive rate, VSMCs’ phenotype, and the expressions of caspase 3 and Bax in normal pulmonary arterial strips treated by DAPT (data not shown). These results suggested that the inhibition of Notch signaling by DAPT in normal pulmonary arteries would not change the biological traits of these arteries.

## Discussion

The rat model of PH inducted by hypoxia is one of the most successful models in the studies of PH. When rats stay in hypoxia environment, the initial contraction of pulmonary arteriole leads to a temporary increase of pulmonary arterial pressure. As the hypoxia persists, the pulmonary arterial remodeling occurs, including thickening of the vessel wall, decreasing the vessel lumen and compliance, and increasing the pulmonary arterial pressure and vascular resistance, ultimately the hypertrophy of right ventricle [3]. In this study, this hypoxia-induced PH was utilized and validated with the body weight decrease and pulmonary arterial pressure increase which reached the peak at 14 days. Consistently, with the prolongation of the hypoxia condition, Fulton index increased in rats. Therefore, the rat model of PH in this study was valid for the evaluation of the roles of Notch system in the pathogenesis of PH.

To date, four Notch receptors (Notch1, Notch2, Notch3, and Notch4) have been defined in mammals. Among them, Notch1, Notch3, and Notch4 exist in blood vessels. In addition, three downstream effectors (HERP1, HERP2, and HERP3) of Notch signaling are mainly present in arteries. In the current study, we examined the localization of Notch receptors in the rat PH model and the results were consistent with previous studies in other conditions: Notch4 is mainly expressed in arterial endothelial cells and occasionally in arterial smooth muscle cells; Notch3 is mainly expressed in arterial smooth muscle cells; Notch1 is expressed in both arterial endothelial cells and smooth muscle cells [9], [13], [14], [16], [17], [18], [21]. However, the expression levels of Notch1, Notch3, Notch4, and the downstream target genes HERP1 and HERP2 all have been changed in pulmonary arteries in the condition of PH. Among them, the mRNA levels of Notch1, Notch3, Notch 4, and HERP1 progressively increased with a peak at one week for Notch 3 and at two weeks for Notch1, Notch 4, and HERP1. The mRNA levels of the four proteins gradually decreased. The time of peaks of mRNA levels were coincident with the time of fastest increase of PH. The results suggested that Notch signaling pathway, especially those factors involved in vascular remodeling, contributed to the development of PH induced by hypoxia. To date, the role that Notch signaling plays in the development of hypoxia induced PH has not been clearly elucidated. But in the studies of neural precursor cells, glioblastoma stem cells, medulloblastoma stem cell, and myogenic cell, it has been found that hypoxia-inducible factor-1α (HIF-1α), an intracellular mediator of oxygen sensing, could interact and stabilize NICD, and activate Notch signaling pathway, to preserve these cell viability and expansion under hypoxic conditions [29], [30], [31], [32]. In addition, HIF-1α and VEGF regulate Notch signaling to accelerate choroidal neovascularization angiogenesis [33]. Thus, NICD is at the convergence point of HIF-1α, which has been implicated in the development of PH. Recently, integration between Notch and BMP signaling pathways has been found in several organs and cell types, while BMPR2 mutations have been found to be associated with the development of familial human PAH [2], [3], [4], [5], [34], [35], [36], [37]. It might be one of the mechanisms that Notch signaling pathway functioned in the development of PH. Especially, as to Notch 3, Li et al showed that the mRNA and protein levels of Notch3 continually increased along with the development of PH in the lungs of idiopathic PH patients as well as in hypoxia-induced PH mice and in monocrotaline-induced PH rats [24]. However, in this study, the mRNA level of Notch 3 reached the peak at one week after hypoxia. This difference may be attributed to the distinct tissues where total RNA were isolated.

In the classic Notch pathway, there are two consecutive protein enzymolysis processes after the conjunction of Notch receptors and ligands. The second enzymolysis involves presenilin-dependent γ-secretase. A similar γ-secretase was found in the transmembrane domain of APP, which is important in the last step of amyloid-β protein production, which is thought to contribute to the pathogenesis of Alzheimer’s disease. DAPT, a small molecular specific inhibitor of γ-secretase, has been shown to have potential therapeutic value for Alzheimer’s disease [38], [39], [40]. Recent studies showed that DAPT could inhibit Notch signaling in Drosophila and cultured cells, such as neuron, nephron cell, and myocardial cell. Furthermore, DAPT can inhibit the translocation of NICD [38], [39], [40]. Because the transduction of Notch signaling is restricted to interaction of ligands and receptors of Notch from cell-to-cell contact, which can be greatly affected by interactions of cells and cellular microenvironments [20], [21]. In the current study, we used an organ culture system containing rat tail collagen [41] to imitate the in vivo environment for vascular adventitia [42], [43]. Of note, because the rat tail collagen is the same vascular extracellular matrix for the pulmonary strip in the culture, it is one key component for successful culturing artery strip to maintain the endothelium, smooth muscle, fibroblasts, and extracellular matrix of the vessel in a similar environment in vivo. Using this system of organ culture, we found the thickness of vascular wall of PH rats decreased 30% after treating with DAPT to the cultured vascular strips of PH rats by hypoxia, as well as the expression level of apoptotic factors (caspase-3 and Bax) increased significantly. The results suggested that DAPT treating could inhibit cellular proliferation and promote apoptosis, which was consistent with previous study [24]. Notably, our results highlighted the bax/caspase-3 pathway in the apoptosis induced by DAPT treating. In addition, while DAPT did not change the mRNA level of Notch receptors of the vascular strips, the mRNA levels of downstream effectors, HERP1 and HERP2, decreased significantly in the presence of DAPT, suggesting that DAPT mediated inhibition of intracellular Notch signaling pathway without impacting on the expression of Notch receptors. At the same time, the mRNA levels of SM-MHC and SM22α increased significantly by DAPT, suggesting a conversion of VSMC phenotype.

Our results suggested an important role of Notch signaling pathway in vascular remodeling in PH, not only Notch3 but also Notch1 and 4. However, this pathway functions differently depending on time, environment, and cell types. Notch system in arterial system is delicately regulated by regulatory factors involved in development and growth factor released following vascular injury [33], [34], [35]. It has been shown that Notch 1 and Notch 3 promoted the proliferation, migration and aggregation of VSMCs, disinhibited cell cycle arrest, inhibited apoptosis of VSMCs, and facilitated the conversion of endothelial cells and fibroblasts to smooth muscle cells [24], [27], [44], [45], [46], [47], [48], [49], [50], [51], [52], [53], [54], [55], [56], [57], [58], [59], [60], [61], [62], [63]. These potential mechanisms may include: 1) inhibiting the transcription of p27kip1 and p21cip1, prompting VSMCs to enter S phase, and causing the proliferation of VSMCs; 2) promoting the transcription of Guanine exchange factor of Rac and Sos1, and leading to increasing activity of Rac1 and proliferation, migration, and aggregation of VSMCs; 3) promoting the expression of C-FLIP, activating Akt, increasing the expression of Bcl-xl and decreasing the expression of Bax, and inhibiting the apoptosis of VSMCs; 4) activating the promoter of Smooth muscle α-actin (SMA) gene, increasing the expression of SMA, and prompting VSMCs to transform to synthetic phenotype and EMT; 5) inhibiting the expressions and activities of various marker genes of VSMCs under certain circumstances, preventing the combination of serum response factor (SRF) and CArG box and differentiation of VSMCs, and prompting VSMCs to transform to synthetic phenotype [24], [27], [44], [45], [46], [47], [48], [49], [50], [51], [52], [53], [54], [55], [56], [57], [58], [59], [60], [61], [62], [63]. In the mean time, Notch 1 and Notch 4 inhibit the proliferation, migration, and apoptosis of endothelial cells, maintain the “quiescence” phenotype of the cells, and keep vascular endothelium stable [64], [65], [66], [67], [68], [69], [70], [71], [72], [73], [74]. Notch 1 regulates endothelial progenitor cell activity [75], [76] and impedes the appearance of endothelial teloblasts and the budding/growth of blood vessels [77]. In summary, the function of Notch pathway in vascular remodeling is complicated. We found that the expressions of Notch1, Notch 3, and Notch4 changed in a time dependent pattern following the development of PH and the inhibition of Notch signaling by DAPT could reverse the biological traits of VSMCs in PH rats, including the decreasing proliferation, the increasing apoptosis, and the phenotypic transformation of VSMCs, finally inhibiting the pulmonary vascular remodeling. Therefore, further investigation is needed to dissect the specific function of each Notch factors, especially Notch1 and 4, in different types of cell and different stages of vascular remodeling in PH.

In conclusion, the current study suggested Notch pathway play an important role in pulmonary vascular remodeling in PH and targeting Notch signaling pathway could be a valuable approach to design new therapy for PH.

## Supporting Information

Figure S1 **The impact of DAPT on Notch signal in vascular strips of normal rats.** (A) the effects of DAPT on the mRNA level of Notch receptors in vessel strips of normal rats. No significant differences of Notch receptors mRNA levels among experimental groups were detected by ANOVA. (B) the effects of DAPT on the mRNA levels of HERP1 and HERP2 in vessel strips of normal rats. The mRNA levels of HERP1 and HERP2 were significantly lower in both low and high dosage of DAPT groups than in blank and DMSO control groups. However, there were no significant differences between blank and DMSO control groups, or between DAPT-low and DAPT-high groups. (*, P<0.05 compared to control group. Data were normalized to GAPDH and expressed as means ± s.e.m.)
(TIF)

Figure S2 **DAPT does not decrease wall thickness in cultured vascular strips from normal rats.** (A-D) Verhoeff iron hematocylin staining of Pulmonary artery vascular strips (×400): (A) pulmonary artery in culturing lung tissue strip treated with additional equivalent M199; (B) pulmonary artery in culturing lung tissue strip treated with DMSO; (C) pulmonary artery in culturing lung tissue strip treated with DAPT (1 µmol/l); (D) pulmonary artery in culturing lung tissue strip treated with DAPT (10 µmol/l). (E) the change of vascular media thickness of hypoxia rats with or without DAPT treating. The vascular media of hypoxia rats treated with DAPT were not significantly different to those treated with M199 or DMSO. (*, P<0.05 compared to control group. Data are expressed as means ± s.e.m.)
(TIF)
