# Supplementary figures and images for: Notch Signaling Change in Pulmonary Vascular Remodeling in Rats with Pulmonary Hypertension and Its Implication for Therapeutic Intervention
Source: PLoS One. 2012 Dec 12;7(12):e51514. doi: 10.1371/journal.pone.0051514 (PMC3520790; doi:10.1371/journal.pone.0051514)

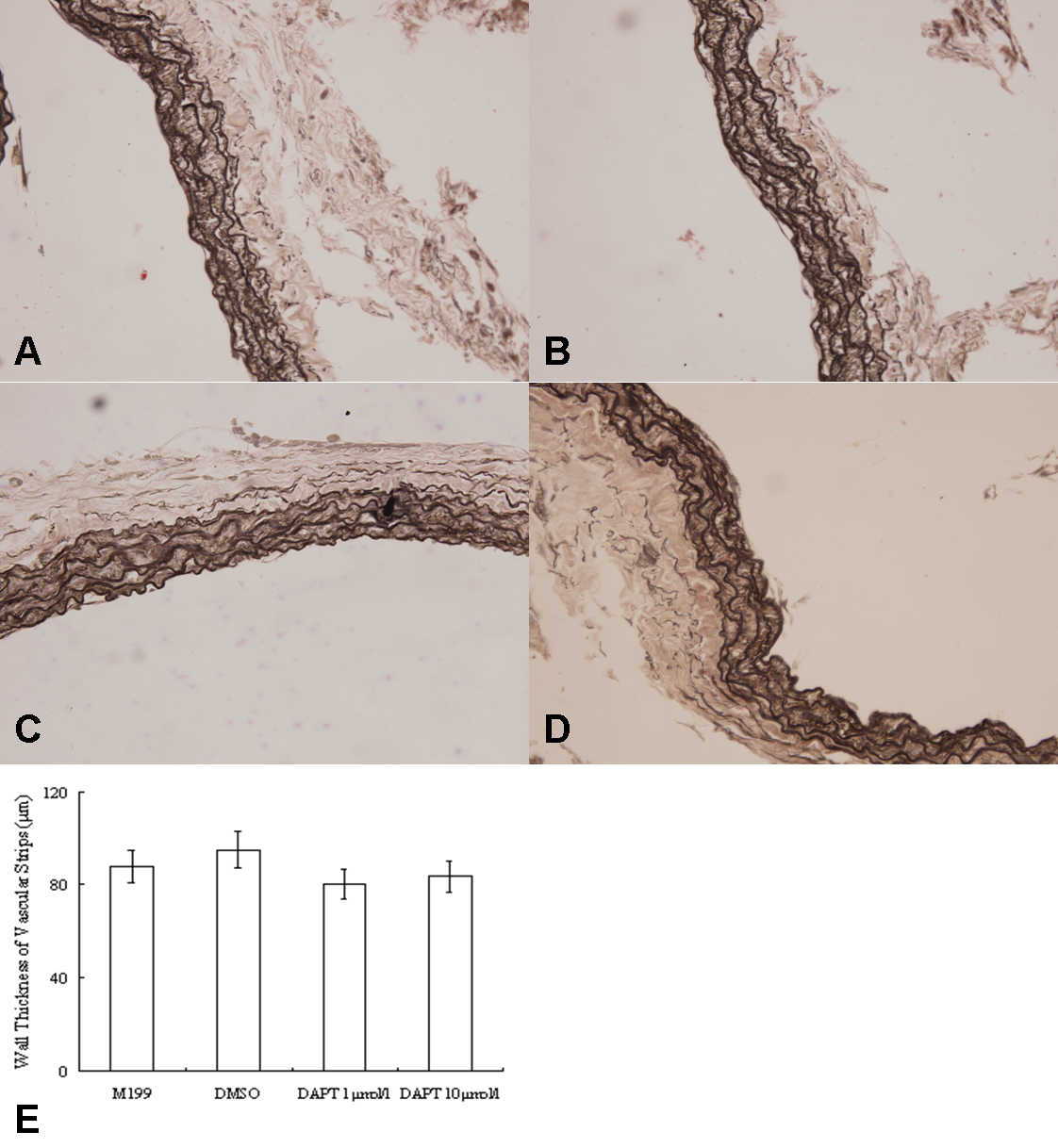

Supplement: Figure S2 — DAPT does not decrease wall thickness in cultured vascular strips from normal rats. (A-D) Verhoeff iron hematocylin staining of Pulmonary artery vascular strips (×400): (A) pulmonary artery in culturing lung tissue strip treated with additional equivalent M199; (B) pulmonary artery in culturing lung tissue strip treated with DMSO; (C) pulmonary artery in culturing lung tissue strip treated with DAPT (1 µmol/l); (D) pulmonary artery in culturing lung tissue strip treated with DAPT (10 µmol/l). (E) the change of vascular media thickness of hypoxia rats with or without DAPT treating. The vascular media of hypoxia rats treated with DAPT were not significantly different to those treated with M199 or DMSO. (*, P<0.05 compared to control group. Data are expressed as means ± s.e.m.) (TIF) [file pone.0051514.s002.tif]
